# Estrogen-Receptor Expression and Function in Female Reproductive Disease

**DOI:** 10.3390/cells8101123

**Published:** 2019-09-21

**Authors:** Zi-Run Tang, Rui Zhang, Zheng-Xing Lian, Shou-Long Deng, Kun Yu

**Affiliations:** 1 Beijing Key Laboratory for Animal Genetic Improvement, National Engineering Laboratory for Animal Breeding, Key Laboratory of Animal Genetics and Breeding of the Ministry of Agriculture, College of Animal Science and Technology, China Agricultural University, Beijing 100193, China; S20183040519@cau.edu.cn (Z.-R.T.); zr123@cau.edu.cn (R.Z.); 2 CAS Key Laboratory of Genome Sciences and Information, Beijing Institute of Genomics, Chinese Academy of Sciences, Beijing 100101, China

**Keywords:** estrogen receptor, ovary, female reproductive disease

## Abstract

Estrogen receptors (ER) include ER alpha, ER beta and new membrane receptor G protein-coupled receptor 30 (GPR30). Estrogen receptors are key receptors to maintain ovarian granulosa cell differentiation, follicle and oocyte growth and development, and ovulation function. The abnormal functions of estrogen, its receptors, and estradiol synthesis-related enzymes are closely related to clinical reproductive endocrine diseases, such as polycystic ovary syndrome (PCOS) and endometriosis (EMS). At present, hormone therapy is the main treatment for ovarian-related diseases, and a stable hormone environment is established by regulating ovarian function. In recent years, some estrogen-related drugs have made great progress, such as clomiphene, which is a nonsteroidal antiestrogen drug in clinical application. This article elaborates on the regulatory role of estrogen and its nuclear receptors and membrane receptors in oocyte development, especially female reproductive diseases related to the abnormal expression of estrogen and its receptors. We also highlighted the latest advances of treatment strategy for these diseases and the application of related targeted small molecule drugs in clinical research and treatment, so as to provide reference for the treatment of female reproductive diseases.

## 1. Introduction

Estrogen mediates various effects throughout the body in both women and men, regulating physiological and pathological processes in the reproductive, cardiovascular, skeletal, endocrine, nervous, and immune systems. Therefore, it is also involved with scores of diseases, for example, infertility, endometriosis, polycystic ovary syndrome, and various cancers. The role that estrogen plays in the female reproductive system and the development of secondary sexual characteristics are primarily recognized as its most significant function. The cellular receptors of estrogen are crucial mediators of estrogen functions, which includes the nuclear receptor family (estrogen receptors (ER) alpha and ER beta) and membrane estrogen receptors (mERs; G protein-coupled receptor 30 (GPR30)). The physiology of estrogen and its receptors is especially complicated, as the history of estrogen-signaling mechanisms and systems originates more than 500 million years ago [1]. Interestingly, it was believed that the actions of estrogen were activated via a single receptor discovered in 1962 [2] until another estrogen receptor with high homology was identified in 1996 [3]. Since then, the former was renamed as ERα and the latter ERβ. The third estrogen receptor was discovered and characterized in the 2000s [4,5], and was named the G protein-coupled receptor 30 (GPR30)/G protein-coupled estrogen receptor 1 (GPER).

Before the discovery of GPER, most physiological functions of estrogen receptors were widely recognized as ligand-activated transcription factors that belong to the nuclear hormone receptor family, including other steroid receptors such as progesterone, androgen, glucocorticoids, and mineralocorticoids [6]. However, this view was proved to be inaccurate by the time frame of transcriptional mechanisms because rapid cellular and physiological responses mediated by estrogen and other steroids had already been demonstrated. GPER identification allowed us to further understand the complicated activities and functions of estrogen and its receptors due to the rapid responses it classically mediates [7,8,9]. As described above, there are multiple and diverse cellular estrogen effects, including cAMP production, calcium mobilization inside cells, and the activation of various kinases, which include phosphoinositide 3-kinase (PI3K) and extracellular signal-regulated kinase (ERK). During these physiological processes, the expression and signaling mechanisms of estrogen receptors is complex and potentially exhibit redundant, independent, synergistic, and/or antagonistic actions [10]. As a result, the abnormal functions of estrogen receptors and estradiol synthesis-related enzymes are closely related to clinical diseases, especially in the reproductive and endocrine systems, such as polycystic ovary syndrome (PCOS) and endometriosis (EMS). Moreover, some numerous estrogenic compounds, including natural and synthetic ligands, are harmful to humans and other animals and were classified into endocrine-disrupting chemicals (EDCs) [11].

Owing to the critical role that estrogen receptors and estrogenic (including antiestrogenic) compounds play in various aspects of health and disease, numerous drugs have been synthesized by pharmaceutical companies, some of which have achieved great success as contraceptives, cancer treatment, and postmenopausal conditions including depression and osteoporosis. Part of these drugs are classified as selective estrogen receptor down-regulators or degraders (SERDs) because their binding to receptors leads to the proteasomal degradation of the receptor [12], while others are sorted into selective estrogen receptor modulators (SERMs) that play a role as agonists or antagonists of estrogen receptors in different types of estrogen-sensitive tissue [13].

As a matter of fact, because of the significant role that estrogen and its receptors play in multiple physiological and pathological processes, hormone therapy is the main treatment for ovarian-related diseases. For example, raloxifene can effectively reduce the incidence of breast and uterine cancer, tamoxifen is now used for the therapy of breast tumors in premenopausal and postmenopausal women, and diethylstilbestrol can be used to treat breast cancer patients. On the other hand, regulation of ovarian function can also maintain a stable hormone environment. In this review, we expound an overview of the regulatory role of estrogen, its receptors, and their cellular mechanisms in the female reproductive system, and recent advances in ovary diseases, followed by research and applications of the latest strategy of clinical therapy, so as to provide reference for the treatment of female ovarian diseases.

## 2. Estrogen Receptors

### 2.1. ERα and ERβ

Estrogen receptors α and β, the critical mediators of the biological effects of estrogen, are encoded by genes *ESR1* and *ESR2,* located on nonhomologous chromosomes, respectively. Moreover, the expression of ERα and ERβ differs greatly in tissue and cells. ERα is predominantly expressed in the uterus, ovaries, and breasts, while expressions of ERβ are mainly found in the nervous system, ovaries, cardiovascular systems, and the male reproductive system [14]. The functions of ERα and ERβ can be targeted by many therapeutic treatments of estrogen-related diseases. Those therapeutic interventions emphasize the significance of understanding the mechanisms of ERα and ERβ to optimize treatment strategy.

Members of the nuclear-receptor superfamily of hormone receptors are constituted by four domains, ERs also included. These four structures and functioning domains are an NH_2_ terminal domain, a COOH terminal ligand-binding domain, a hinge region, and a DNA-binding domain [15]. In addition, people found that ERs have a different domain with an unknown function: the carboxyl-terminal domain [16]. When we compared all of these five domains of ERα and ERβ, we found that the NH2-terminal domains encompassed a ligand-independent activation function (AF1) domain that is capable of transcriptionally activating target genes with less than 20% similarity between the two forms. The amino acid identity of the DNA-binding domain in ERα and ERβ is 97%, and this domain mainly acts as a mediator of the specific combination of ERs and DNA sequences [17]. On the contrary, the amino acid identity of the ligand-binding domains of ERα and ERβ is 59%, with only minor structural differences between the two subtypes of ligand-binding pockets [18]. Moreover, the COOH terminal ligand-binding domain contains the transcription activation function 2 (AF2) domains, which are critical for the regulation of ligand-dependent transcription [19].

To mediate the effects of gene expression, nuclear hormone receptors are localized in the nucleus. As for estrogen receptors, inactivated ERs are mainly located in the nucleus (~95%), and the rest are located in the cytoplasm, and the membrane-localized ERα acts as an atypical G-protein coupled receptor [20,21]. Ligand activation usually leads to the dimerization of the monomer after dissociation from the chaperone (Hsp90), and translocate cytosolic receptors to the nucleus. Like other steroid hormone receptors, ERs act as homologous dimers and/or heterodimers in transcription, recruiting coregulators and combining them with estrogen response elements (EREs) [6,22,23]. Through combining with transcription factors, activated estrogen receptors can also indirectly bind to DNA, and transcription can be regulated by post-translational modification in the absence of a ligand [24]. A variety of ligands that are able to bind to the ER produce various conformations of the binding domain of the receptor-ligand (particularly helix 12), which produces numerous binding sites for coregulatory factors and other proteins. Furthermore, differential expression modes in different types of tissue lead to the complexity of ER and its ligands [6]. In summary, ERs regulate the expression of numerous genes, both positively and negatively, which depends on ligand and tissue [25].

### 2.2. G Protein-Coupled Estrogen Receptor

GPER was originally identified in the laboratory as an orphan receptor (a cloned receptor with no known ligand) in the late 1990s and was named GPR30, which belongs to the 7-transmembrane G protein-coupled receptors family (GPCR) [26]. The vast majority of the available data illustrated that GPR30 specifically binds to estrogen and activates the intracellular signaling cascade normally associated with GPCR. That is the reason why GPR30 was designated as a GPER [27,28]. The transmembrane domains of the GPCR positions the amino terminus outside the cell, which is typically glycosylated; the carboxyl-terminal is in the cytoplasm and has great effects on receptor internalization through phosphorylation [29]. At present, the positioning of GPER is still controversial. Using both cell and tissue samples, most studies have resulted in consistent results with mainly the cytoplasmic membrane localization of GPER [30,31,32], while localization in the nucleus has been observed, too [33,34].

GPER is coupled with a heterotrimeric G protein that, in turn, regulates multiple downstream intracellular effectors. The most active natural form of estrogen is 17β-estradiol (E2), which is formed predominantly in the ovaries of premenopausal women. GPER was demonstrated to be involved in the rapid activation of ERK1/2 mediated by 17β-estradiol [35,36,37]. The downstream signaling pathway is involved in the activation of metalloproteinase mediated by Src, which releases heparin-binding EGF, followed by the transactivation of EGFR and then ERK1/2. After that, the capability of GPER to activate adenylate cyclase was illustrated as a mechanism involved in the activation and/or inhibition in ERK1/2 [38]. On the flip side, GPER activates the PI3K/Akt pathway and responds to 17β-estradiol with EGFR transactivation involved. In addition, 17β-estradiol can stimulate the growth of human keratinocytes by inducing cyclin D2 expression, and induce the expression of c-Fos (a cyclic adenosine monophosphate signal in macrophages) to enhance the production of nerve growth factor, which are both mediated via cell surface GPER [39,40]. In spite of rapid signaling events, GPER also regulates gene expression, such as c-fos and D1 [41,42], growth factor of connective tissue [43], fatty acid synthase [44], and vascular endothelial growth function [45]. All of these different GPER activation pathways regulate a variety of cellular physiology functions in proliferation, metabolism, migration, and secretion, which also shows the great influence of GPER in physiological and pathological processes. As mentioned above, ERα and ERβ mediate multiple physiological processes. At the cellular level, ERα, ERβ, and GPER may function synergistically or antagonistically, which means the final cellular outcome would be decided by the interaction of all activation and inhibition pathways.

### 2.3. Estrogen Receptor Ligands

**Figure 1:**
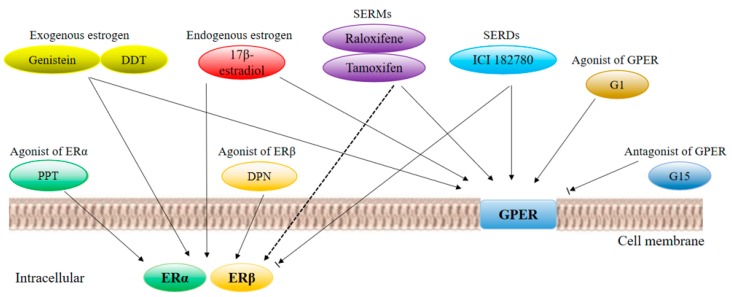
G-protein-coupled estrogen receptor (GPER) and agonists and antagonists of cell-membrane-bound receptors derived from estrogen receptor (ER)α and ERβ, involved in rapid intracellular signal transduction. Arrows indicate activation, blocked arrows indicate inhibition, and dashed lines indicate tissue-specific activation or inhibition.

Estrogen receptor ligands and other factors affect the hormone signal transduction pathway: The modulation of endogenous ligands and coregulators can be a candidate for tumors and therapeutic targets. In addition to 17β-estradiol, estrogen receptor ligands can activate the receptors of each subtype, and the synthesis and use of specific ligands have received extensive attention. These compounds, SERMs, bind to these receptors with specificity in transcriptional-activation activity (for example, tamoxifen is an ERα-selective agonist or antagonist, and raloxifene is an ERβ-selective agonist or antagonist). In order to study the molecular mechanisms of the action of three estrogen receptors, many synthetic selective agonists or antagonists have been successfully developed: 4,4′,4′’-[4-propyl-(1H)-pyrazole-1,3,5-triyl] trisphenol (PPT) activates ERα, 2,3-bis (4-hydroxyphenyl)–propionitrile (DPN) is an agonist of ERβ, and compound G-1 is a highly specific agonist of GPR30. Figure 1 shows various estrogen receptor ligands, including endogenous and exogenous ligands, SERMs, SERDs, and GPER agonists and antagonists, which correspond to their receptors.

### 2.4. Cell Mechanisms

**Figure 2:**
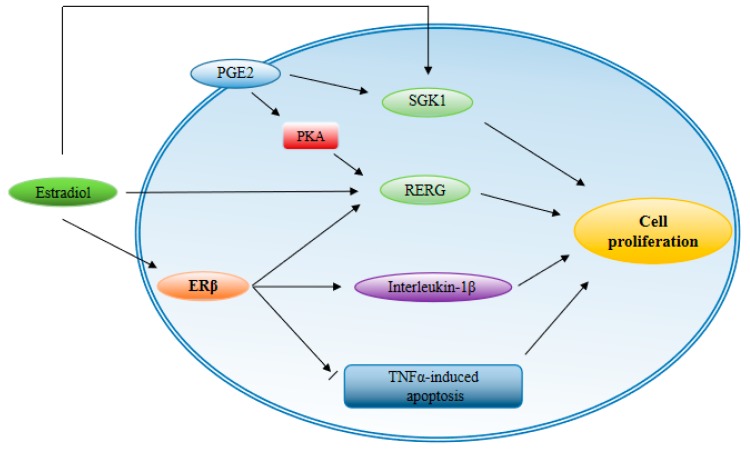
Expression of ERβ in endometriosis. ERβ mainly participates in endometriosis through the following ways: Inhibiting TNFα-mediated apoptosis, inducing an increase in interleukin-1, and co-stimulating Ras-related and estrogen-regulated growth inhibitor (REGE) expression with prostaglandin E2 (PGE2) under the action of estradiol. In addition, serum and glucocorticoid-regulated kinases (SGK1) is a co-targeting target of PGE2 and estradiol. These pathways lead to proliferation of endometriosis cells.

Estrogen mediates its biological response through several possible cellular mechanisms, as shown in Figure 2. There are two main cellular roles involving receptors: genomic activity and rapid nongenomic effects [46]. It was reported that the rapid effects take place within minutes in the process of therapy. Furthermore, by inhibiting the MAPK/ERK or AKT signaling pathway, nongenomic effects can be stopped. Initiating the processes of these signaling pathways is closely related to GPR30-mediated plasma-membrane-associated processes [47]. Here, we elaborate on the process of ERα transportation to the cell membrane and the signaling that follows. ER can bind to caveolin 1 (Cav-1) physically induced by ERα palmitoylation [48]. After the binding process, Cav-1 transports ER to the caveolae rafts in the membrane and is capable of assembling various signaling molecules [49]. The activation process of ER is achieved by association with G-protein α and β/γ subunits after its linking with E2 and the following dimerization [50,51]. The activation of the G-protein can initiate multiple rapid nongenomic effects in a matter of seconds, including cyclic nucleotide production and early kinase activation. On the other hand, because of signaling to the epigenome of membrane ER and the improvement of receptor activity caused by nuclear ER protein post-translational modification, non-palmitoylated nuclear-localization ER is recruited to promoters and enhancers that regulate genes required for the regulation of steroid action. During the binding of hormones to extranuclear receptor proteins, the steroid-binding unit of the protein changes, which is called receptor transformation. Receptor transformation is an important step in the action of estrogen, and hormone-induced receptor proteins are converted into biochemically functional forms [52].

There are three predominant mechanisms of genomic transcriptional regulation mediated by estrogen receptors. The classical mechanism (direct binding to DNA regulatory elements) can be illustrated by the following example. Helix 12 is the functional core of AF-2 and is highly conserved in ligand-binding domains. The process of binding to a ligand can alter the configuration of helix 12, which leads to an agonistic or antagonistic form of transcriptional regulation [53]. Binding between ER and hormone results in a change of conformation in the ligand-binding domain that allows helix 12 to interact with coactivators. The resulting genomic reaction requires coactivator binding and is proportional to the magnitude of the reaction. On the other hand, as shown by studies at the cellular level and the living individual level, ERα binds to the DNA with inactive status in the absence of hormones [54,55]. A mouse model of DNA-binding domain mutation of ERα indicates that direct DNA binding is required to activate both biological activity and hormone response. Nuclear factors such as pro-factor FoxA1 may affect direct binding, and by recruiting chromatin at the binding site to remodel the protein, the chromatin is opened, allowing the ER to enter its regulatory DNA site. Following this, the recruitment of polymerase II initiates gene transcription after assembling the transcription complex, which is comprised of multiple components [56]. In the second ER-regulated mechanism, ER binds to transcription factors that are already bound to the DNA. Then, hormone receptors regulate gene expression first via the interaction between proteins and transcription factors, then, these transcription factors directly bind to their respective response elements [57]. As for the third mechanism, ER can regulate hormone responses without hormones through the activation of growth factors without ligands, which is attributed to the phosphorylation process of some serine residues on the receptor [58]. In addition to its ability to directly regulate gene expression, estrogen also affects cell signaling and cellular function through rapid membrane-initiation events. Many signaling processes rely on estrogen receptors localized to the plasma membrane. Lipid rafts are critical for ER plasma-membrane localization and play a key role in its membrane-priming effect [59]. Together, the integration of these cellular signaling pathways can mediate genomic activities and rapid nongenomic effects independently and/or complementarily, which activates the effects of estrogen through hormonal response.

## 3. ERs and Female Reproductive Diseases

### 3.1. ERs and Ovarian Cancer

In women of childbearing age, ERα is mainly located at thecal cells and the ovarian stroma in the corpus luteum and surface epithelium of the ovary. In postmenopausal women, ERα is present in the stroma, the epithelial inclusion cyst, and the ovarian-surface epithelium. The main locations of ERβ are granulosa cells. Growths that depend on estrogen in response to endocrine therapy for ovarian cancer are closely related to the expression level of ERα. ER is downregulated in ERα-positive ovarian cancer, while being targeted directly by tumor suppressor microRNA (miR)-206. Therefore, the introduction of miR-206 mimics could inhibit cell proliferation and the invasion of ovarian-cancer cells [60,61]. Recent studies have shown that long noncoding RNAs can mediate the function of ERα in ovarian cancer [62]. The promotion of ERα in ovarian cancer suggests that endocrine therapy may be an efficacious option. Unfortunately, ovarian cancer is not often treated with antiestrogenic drugs because of the low response rates. As ovarian cancer occurs, the ratio of ERβ and/or ERβ/ERα decreases, indicating that carcinogenesis may be associated with the loss of ERβ expression. Treatment or reintroduction of ERβ with ERβ agonist DPN significantly inhibits the growth of ovarian cells [63]. It is worth noting that recent studies have shown that a normal ovarian epithelium almost completely shows ERβ cell-nuclear positive immunity, while ovarian-cancer tissue mostly shows the cytoplasmic staining of ERβ. Therefore, the expression of cytoplasmic ERβ is recognized as an independent unfavorable prognostic factor [64]. In addition, statistical data indicate that cytoplasmic ERβ2 (one of the isoforms of ERβ) expression is positively correlated with five-year survival and decreased chemical resistance [65]. These new results suggest that different isoforms of ERβ may function in different ways, which is possibly due to their different cellular localization and prognosis. Recent studies have shown that natural ERβ agonists have the potential to significantly inhibit the growth of ovarian-cancer cells through anti-inflammatory and proapoptotic effects, and can be used as novel therapeutic agents for the treatment of ovarian cancer [66].

### 3.2. ERs and Endometriosis

Endometriosis refers to a common gynecological disease formed by the active endometrial cells being implanted outside the endometrium [67]. The symptoms have a negative impact on the health and quality of life of the patient. Among all patients with endometriosis, according to statistical data, 40%–50% have fertility problems [68].

Estrogen-mediated changes in cell signaling have important implications for the pathogenesis of endometriosis. The invasion and migration of endometriosis eutopic endometrial stromal cells (euESC) can be regulated by estrogen/H19/miR-216a-5p/ACTA2 pathways. Specifically, the invasion and migration of euESC can be inhibited by the suppression of H19 or ACTA2, and promoted by estrogen via H19 [69]. Endometriosis contains higher levels of 17β-estradiol than the normal endometrium because of higher levels of aromatase and 17β-hydroxysteroid dehydrogenase-1 genes [70]. High levels of 17β-estradiol activate ER in endometriotic tissue and stimulate growth that depends on estrogen. In endometriotic tissue, both the ERα and the ERβ isoforms are required for the growth of endometriotic lesions. In mice, ERα knockout females are sterile because their uteruses are not sensitive to estrogen. On the contrary, ERβ knockout females are sub-fertile and primarily lack effective ovulation function [71]. The important role of ERα and ERβ in the development of mouse ectopic lesions have been revealed in previous research [72,73]. Functional studies on ERβ have shown that it can prevent apoptosis, enhance adhesion, invasion, proliferation, inflammatory body activity, and inflammatory signals of ectopic lesions. Studies on ERα knockout mice with endometriosis have shown that ERα brings cell adhesion and proliferation, and regulates inflammatory signaling in ectopic lesions [74]. However, different isoforms of ER mean various kinds of expression patterns between types of tissue with endometriosis and a normal endometrium [75]. Contrary to the case of ERα, not only can high levels of estrogen receptors be detected in tissue with endometriosis, but also enhanced ERβ activity. In addition, ERβ-selective antagonists promote the inhibition of ERβ activity and inhibit the growth of ectopic lesions in mice. It is worth noting that the acquisition of ERβ function stimulates endometriotic processes. ERβ inhibits TNFα-induced apoptosis through interactions with apoptotic mechanisms to avoid the endogenous immune surveillance of survival cells [76]. ERβ also promotes the expression level of interleukin-1β through interactions with components of the cytoplasmic inflammatory body, thereby enhancing its cell-proliferation characteristics. In order to identify the target of ERβ in endometriosis, a genome-wide comparative analysis method was used to identify Ras-related and estrogen-regulated growth inhibitor (RERG) and serum and glucocorticoid-regulated kinases (SGK1) [77,78]. Among them, RERG can induce the proliferation of primary endometriotic cells, and the levels of RERG mRNA and protein in human endometriotic stromal cells can be induced by estradiol. Prostaglandin E2 (PGE_2_) can also phosphorylate RERG. Thus, integration of ERβ and PGE_2_ signaling in RERG results in endometrial ectopic cell proliferation. In addition, since the expression of SGK_1_ is stimulated by estradiol and ERβ, the number of endometrial ectopic cells is increased [79]. ERβ functions in a variety of ways to promote cell-proliferation and tissue-invasion activity in endometriosis sites to establish ectopic lesions (Figure 2).

GPER expression in mature follicles/oocytes is more frequent than in primordial follicles/oocytes, which means that GPER may be the choice during follicular development. In addition, GPER is upregulated in ovarian endometriosis [80]. GPER is maximally expressed during the proliferative phase. There is overexpression of GPER in the eutopic and ectopic endometrium in patients with endometriosis compared with the eutopic endometrium of normal participants [81]. In addition, recent research has found and optimized pyridyl-cycloalkyl-carboxylic acids as an inhibitor of microsomal prostaglandin E synthase-1 for the treatment of endometriosis [82].

### 3.3. ERs and Polycystic Ovary Syndrome

Polycystic ovary syndrome (PCOS) is a complex endocrine and metabolic abnormality, common in women of childbearing age, and is the most common female endocrine disease. It is characterized by chronic anovulation (ovulation dysfunction or loss) and hyperandrogenism (excessive male-hormone production in women). The main clinical manifestations are an irregular menstrual cycle, infertility, hairiness, and/or acne [83,84]. PCOS is associated with global and gene-specific DNA methylation remodeling in a cell-type-specific manner [85]. Furthermore, PCOS is an important risk factor for type 2 diabetes, cardiovascular disease, gestational diabetes, pregnancy-induced hypertension, and endometrial cancer [86]. Studies have shown that the endometrial phenotype and dysfunction of women with PCOS are abnormal. PCOS women are at least three times more likely to develop endometrial cancer (EC) [87,88]. Endometrial cancer is associated with endometrial hyperplasia, no antagonistic estrogenic effects and genetic alterations, and is classically classified into two types: type I, which is the most prevalent and estrogen-dependent, and type II, which is more aggressive and generally considered to be estrogen-independent.

In a normal menstrual cycle, the life cycle of the endometrium is divided into a proliferative phase and a secretory phase. It is based on the response of the endometrium to steroid hormones (progesterone, androgen, and estrogen) up- or down-regulated [89]. The main cell types that steroid hormones target in the endometrium are epithelial cells and stromal cells. The activity of mitosis and extensive proliferation activated by estrogen are two main characteristics of the proliferative phase, and elevated estrogen promotes expression levels of ERα and ERβ, which are the highest in the late phase of proliferation [90,91]. Estradiol increases the expression of progesterone receptors mainly through ERα activation, thereby causing the action of progesterone on the endometrium, and triggering the secretory phase of endometrial circulation. In contrast, progesterone inhibits ER expression on the endometrium and inhibits estrogenic effects, thereby initiating endometrial reprogramming and inducing interstitial differentiation. In recent years, studies have demonstrated that several endometrial features associated with the PCOS phenotype may explain the clinical manifestations that are related to an adverse endometrium. ER is the most prominent endometrial marker in women with PCOS. A study showed that expression of ER was increased in the stroma and glandular epithelium of PCOS women when compared to a normal endometrium [92,93]. However, in two relatively advanced studies, gene-expression differences between female endometrial samples in PCOS and control groups were reported, and no changes in ER expression were found [94,95]. Studies have also shown that the expression of p160 steroid receptor coactivator is increased in the endometrium of PCOS women, which may promote the activation of ERα and regulate estrogen effects [92,93]. In short, an abnormal steroid environment may alter endometrial receptivity in these women.

At present, drug treatment of PCOS has replaced surgical treatment as the preferred treatment, and the treatment route is mainly related to the patient’s fertility requirements. PCOS can be treated by reducing hyperandrogenism by using oral contraceptives, glucocorticoids, spironolactone, and fluorinated amides. PCOS patients with fertility requirements need to use ovulation-induction therapy to prevent pregnancy. There has been great progress on drugs for ovulation induction in the past 50 years. Clomiphene is the first choice, which can bind to the hypothalamic estrogen receptor, causing the central nervous system to block estrogen levels in circulation, and pulsed GnRH and gonadotropin secretion are increased, further causing follicular growth and development. In addition, clomiphene can directly affect the pituitary and ovaries, respectively, increase gonadotropin secretion, synergistically enhancing FSH-induced aromatase activity. Clomiphene also exhibits antiestrogenic properties in other parts of the female reproductive tract, especially the endometrium and cervix.

In adolescent populations, the pathophysiology of PCOS is multifactorial. Medical therapy includes combined hormonal contraceptives spironolactone and metformin [96], oral contraceptive pills, and local treatments for hirsutism and acne [97]. The latest research showed that aromatase expression may be affected by epigenetic modifications and the binding of differential ERβ to the proximal CYP19A1 promoter, possibly involved in enhanced aromatase transcription during ovarian stimulation in PCOS patients [98].

## 4. Therapeutic Drugs

**Figure 3:**
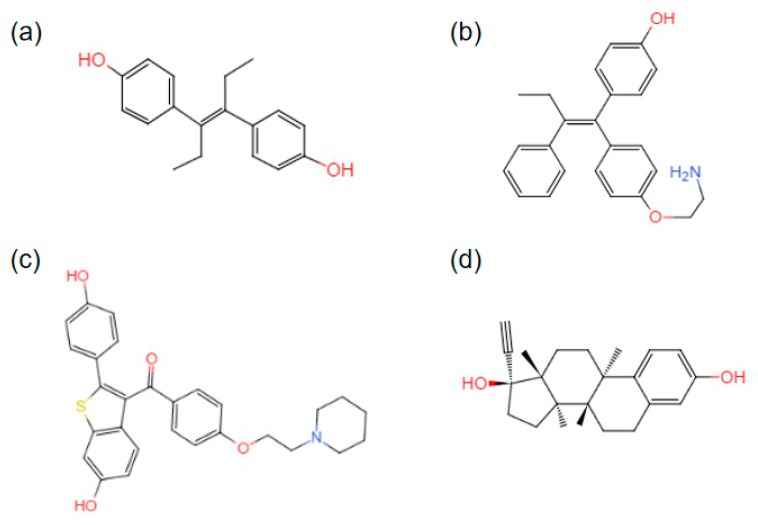
Structures of synthetic therapeutic drugs: (**a**) diethylstilbestrol; (**b**) 4-hydroxytamoxifen; (**c**) raloxifene; and (**d**) ethynylestradiol.

In current therapeutic strategies, a variety of nonsteroidal compounds (with agonist or antagonist activity) and synthetic estrogen derivatives have been developed corresponding to different clinical symptoms and needs. As mentioned above, the ligand-binding domains of ERα and ERβ show a high level of homology, but tissue distribution and the physiological effects are very different. The discovery of GPER further complicates ligand-binding selectivity, affinity, and differences in the types of responses mediated by estrogen-receptor subtypes and classes. Successfully researched and developed selective agonists and antagonists make it easier to distinguish between subtypes of ERα, ERβ, and GPER. Moreover, this has provided a more powerful tool for research and has optimized existing treatments. Some examples are shown below (Figure 3).

The simple structure of the phenolic stilbene compound diethylstilbestrol (DES) has a binding affinity for ERα/β that is about four times higher than that of E2 [99]. The selective ERα/β of DES has been used in research to distinguish between ERα/β-mediated biological responses.

Tamoxifen was originally developed for contraceptive use, but is now used as adjuvant antiestrogen treatment for the therapy of early and late ERα-positive breast tumors in premenopausal and postmenopausal women [100].

Raloxifene (Evista) is a phenolic benzothiophene benzoketone substituted with an alkaline piperidin-1-ylethoxy side chain that acts as a SERM relative to ERα/β. Raloxifene can elicit estrogen on bone; this characteristic leads to the treatment of osteoporosis in postmenopausal women, and the antiestrogen effects of raloxifene in breasts and the uterus have been shown to effectively reduce the incidence of breast cancer, and reduce uterine-cancer and thrombosis risk. Raloxifene exhibits strong GPER efficacy as a GPER agonist [101].

17α-ethynyl-17β-estradiol (also known as ethinyl estradiol) is a synthetic estrogen used in oral contraceptives because of its prolonged biological activity in vivo, and ability to reduce the metabolism. The affinity of ethinyl estradiol for ERα/β is approximately twice that of 17β-estradiol [102].

## 5. Concluding Remarks

This article details the regulation and mechanisms of estrogen, and its nuclear and membrane receptors, the latest advances in diseases caused by the abnormal expression of estrogen and its receptors in the ovary, and related targeted therapeutic drugs applied in clinical research and treatment. Estrogen and its receptors play a key role in the pathophysiology of various systems in the human body. In this review, we highlighted their mechanisms of action and related diseases in the female reproductive system (mainly ovarian cancer, PCOS, and EMS). Research on these three ER-related diseases is still ongoing, and some of the latest research results also reveal the pathophysiology of the disease step by step. Based on the ongoing research progress, more clinical drugs with more effective and minimal side effects will be developed and applied. Studies of estrogen receptors have been ongoing for more than 50 years, and this brief review can only provide a measurable overview of the broad knowledge of these receptors. We hope to provide a reference for the treatment of female ovarian diseases.
